# High-Density Lipoprotein Cholesterol, Obesity, and Mammographic Density in Korean Women: The Healthy Twin Study

**DOI:** 10.2188/jea.JE20100078

**Published:** 2011-01-05

**Authors:** Joohon Sung, Yun-Mi Song, Jennifer Stone, Kayoung Lee, Sun-Young Kim

**Affiliations:** 1 Department of Epidemiology and Institute of Health and Environment, School of Public Health, Seoul National University, Seoul, Korea; 2 Department of Family Medicine, Samsung Medical Center, and Center for Clinical Research, Samsung Biomedical Research Institute, Sungkyunkwan University School of Medicine, Seoul, Korea; 3 Centre for Molecular, Environmental, Genetic and Analytic Epidemiology, University of Melbourne, Melbourne, Australia; 4 Department of Family Medicine, Busan Paik Hospital, Inje University College of Medicine, Busan, Korea; 5 Seocho Samsung Clinic, Kangbuk Samsung Hospital, Sungkyunkwan University School of Medicine, Seoul, Korea

**Keywords:** breast cancer, high-density lipoprotein cholesterol, mammography, obesity

## Abstract

**Background:**

High-density lipoprotein cholesterol (HDL-C) is reported to be associated with breast cancer risk. To better understand this association, we examined the relationship between HDL-C and mammographic density, a putative intermediate risk factor for breast cancer.

**Methods:**

The study subjects were 711 Korean women from the Healthy Twin study. Lipid parameters were assayed enzymatically in fresh sera, and percent dense area (PDA) and absolute dense area were measured from digital mammograms using a computer-assisted method.

**Results:**

PDA was positively associated with HDL-C in both premenopausal and postmenopausal women in a multivariable-adjusted linear mixed model, but the association did not persist when the model was additionally adjusted for body mass index (BMI). BMI was inversely associated with PDA, and this association did not change after additional adjustment for any lipid parameter. Multivariable-adjusted analysis showed that there were significant additive genetic cross-trait correlations between PDA and both HDL-C (coefficient, 0.175) and triglyceride (coefficient, −0.262). However, those correlations disappeared after additional adjustment for BMI.

**Conclusions:**

HDL-C alone is unlikely to increase the risk of breast cancer in Korean women, particularly through changes in breast parenchyma that are apparent in mammographic density. BMI should be included in studies using analytical models where mammographic density is used as an intermediate risk factor for breast cancer.

## INTRODUCTION

High-density lipoprotein cholesterol (HDL-C) has been reported to be associated with the risk of breast cancer,^1^^–^^3^ which may be due to its effect on cell proliferation in the breast.^4^^,^^5^ However, studies of this association have reported inconsistent results: some noted that high HDL-C increased the risk of breast cancer,^1^^,^^6^^,^^7^ whereas others reported that lower levels of serum HDL-C were associated with an increased risk of breast cancer.^2^^,^^8^^–^^12^ In one study, significant associations were observed only in specific groups, such as overweight or obese women.^2^ Another study found no association between HDL-C and breast cancer.^13^

Mammographic density has been positively associated with breast cancer,^14^^–^^17^ and similar associations of mammographic density with known reproductive risk factors for breast cancer have been found consistently in both white and nonwhite female populations.^18^^–^^20^ These findings support the view that mammographic density might be useful as an intermediate marker of breast cancer risk.

Because lipid levels are correlated with endogenous sex hormone levels^3^^,^^21^ that may be involved in breast stromal proliferation, the existence of an association of HDL-C with mammographic density would provide needed insight regarding the association between HDL-C and breast cancer and the underlying biological pathway. A few studies have examined the association between HDL-C and mammographic density,^22^^,^^23^ with inconsistent results. Boyd et al^22^ reported a positive association, but this association disappeared after adjustment for a wide range of covariates, including known risk factors for breast cancer, lifestyle factors, apolipoprotein (Apo) A, and Apo B. Another study found no evidence of an association between mammographic density and HDL-C.^23^ Clearly, further investigation is necessary.

Obesity is closely related to serum lipid level and breast cancer. Obesity increases the risk of breast cancer in postmenopausal women through a hormonal mechanism involving the metabolism of an androgenic precursor to estrogen in adipose tissue,^24^^,^^25^ and estrogen influences breast parenchyma proliferation.^20^^,^^26^^–^^28^ Thus, obesity may be involved in the associations of HDL-C with mammographic density and breast cancer.

In this study, we examined the relationship between mammographic density and HDL-C and other lipid parameters in obese and nonobese women. We also examined the effect of menopausal status on this relationship, since the associations between breast cancer and some risk factors, such as obesity, often differ according to menopausal status.^24^ In addition, we investigated the extent to which any correlation between mammographic density and lipids could be explained by similar genetic or environmental factors in obese and nonobese women. To our knowledge, no study of the association of HDL-C with mammographic density or breast cancer has been conducted in Asian women, who differ from Western women in HDL-C level,^29^^,^^30^ mammographic density,^31^^,^^32^ and risk of breast cancer.^33^^,^^34^

## METHODS

### Participants and study description

The study participants were female members of the Healthy Twin study who had received a mammogram and had lipid profile data collected during a routine health examination. The Healthy Twin study is an ongoing nationwide community study conducted in 3 centers in Korea (Samsung Medical Center, Pusan Paik Hospital, and Dankook University Hospital), and participants were voluntarily recruited without any ascertainment of health status, including breast diseases. Details regarding the overall methodology of the Healthy Twin study have been previously published.^35^ A total of 2278 Korean adults (881 men and 1397 women; age, ≥30 years) comprising twins and their first-degree family members were recruited for the Healthy Twin study from April 2005 through December 2007. A mammogram was obtained for female participants who were 40 years or older at the time of participation in the study or were willing to undergo screening mammography. Among the 734 women who underwent mammography, 23 were excluded from this study: 4 because genetic tests indicated that they were unrelated to their family and 19 because they were taking lipid-lowering medications. Thus, 711 women were ultimately included in the analysis: 113 pairs of monozygotic twins, 30 pairs of dizygotic twins, and 425 singletons.

### Mammographic and clinical variables

Mammograms were taken using a full-field digital mammography system (Senographe 2000D/DMR/DS; General Electric, Milwaukee, WI, USA). A single observer took measurements from 1 craniocaudal view of the right breast for each woman, using a computer-assisted thresholding technique called Cumulus, in which the total area of the breast and the area of dense tissue are measured and the percent dense area is calculated. This measure has been shown to be highly reproducible and reliable.^36^ Mammograms were randomized first by family into reading sets of approximately 100, thereby ensuring that all twins and relatives of the same family were measured in the same set. The reader was blinded to all indentifying information, and a 10% random sample of repeats was included in each set and between every third set to test the reliability of the measurement.

Blood samples were drawn on the same day when the mammogram was obtained, after a 12-hour overnight fast. Serum concentrations of total cholesterol (T-C), HDL-C, triglyceride (TG), and low-density lipoprotein cholesterol (LDL-C) were assayed enzymatically in fresh sera using commercial kits in a designated central laboratory.

Weight (kg) and height (cm) were measured in light clothing using standardized scales and stadiometers. Body mass index (BMI) was calculated as the weight divided by the height squared (kg/m^2^). The use of standardized protocols and training of research coordinators and research assistants ensured that all procedures for anthropometric measurement were consistent between centers.

A self-administered questionnaire was used to collect information about health behaviors (smoking, alcohol consumption, and physical activity), reproductive history (age at menarche, age at first childbirth, number of live births, duration of breast feeding, menopause, use of oral contraceptives, and use of hormone replacement therapy), and medical and demographic characteristics (age, education level). An additional face-to-face interview was conducted by a trained interviewer to clarify incomplete or ambiguous responses. Postmenopausal status was defined as no menstruation for more than 12 months. If a woman underwent hysterectomy but her ovarian function was unknown, she was classified as postmenopausal only if she had received estrogen replacement therapy or was 55 years or older.

Zygosity of twin pairs was identified by 16 short tandem repeat (STR) markers (15 autosomal STR markers and 1 sex-determining marker) in 67% of twin pairs. In the remaining 33% of twin pairs, zygosity was determined by a self-administered zygosity questionnaire that was validated as being 94.3% accurate in an STR marker study (manuscript submitted for publication).

All participants provided written informed consent when they visited one of the study centers. The study protocol was approved by the Korea Center for Disease Control and the institutional review boards of the 3 participating centers.

### Statistical analyses

Intra-class correlation coefficients were estimated to assess reliability of repeated measurements of mammographic density within reading sets in 65 randomly chosen women. Age-adjusted residuals of each of the mammographic measures were inspected for normality, and log transformations were applied to both of the mammographic measures.

Because lipid parameters are closely related with age, we used analysis of covariance to calculate age-adjusted levels for these parameters according to the distribution of each selected characteristic and breast cancer risk factors, and the linear trend was examined using age-adjusted linear regression analysis.

Associations of lipid parameters with mammographic density measures were evaluated using a linear mixed model in which household and twin effects were adjusted as random effects, and other covariates (age, smoking, alcohol consumption, physical exercise, education level, number of live births, age at birth of first child, duration of breast feeding, oral contraceptive use, menopausal status, and use of hormone replacement therapy) were adjusted as fixed effects.

We examined the effect of menopausal status on the relationship between mammographic density and lipid parameters by repeating the linear mixed model separately in premenopausal women and postmenopausal women, and by testing the statistical significance of interaction terms (a product of lipid profile categories and menopausal status category).

We examined the effect of obesity (measured by BMI) on the relationship between mammographic density and lipid profile by repeating the linear mixed model, with and without adjustment for BMI, and by repeating the analysis for obese (BMI ≥25) and nonobese (BMI <25) women. The cut-off level for obesity was determined according to the obesity guidelines for Asian-Pacific populations.^37^

To collect evidence of common genetic regulation between mammographic measures and lipid parameters, we conducted bivariate analysis to partition the phenotypic correlations into genetic (ρ_G_) and environmental correlations (ρ_E_) using SOLAR (Sequential Oligogenic Linkage Analysis Routines) version 2.0.^38^ A significant genetic correlation was considered evidence of pleiotropy. To estimate independent genetic correlations with respect to obesity and other covariates, we adjusted first for age, then other covariates (except BMI), and, finally, BMI.

## RESULTS

When we estimated the reliability of mammographic density measurement, the correlation coefficients between repeated measurements for total area, dense area, and percent dense area were 0.99, 0.98, and 0.98, respectively.

Table 1 shows age-adjusted levels of lipid parameters according to the distribution of selected participant characteristics. HDL-C was progressively higher with increasing percent dense area, whereas LDL-C and TG were lower. Dense area was not associated with levels of HDL-C, LDL-C, or TG. HDL-C was lower with increasing BMI, while LDL-C and TG progressively increased. HDL-C was higher with increasing educational attainment, while TG was lower. HDL-C was higher among ever-smokers, alcohol drinkers, women with fewer living children, and women who had breastfed their infants for a shorter time.

**Table 1.** Age-adjusted means (SE) for lipid parameters, according to selected participant characteristics and risk factors for breast cancer

Characteristic		No.	Total cholesterol (mmol/L)		HDL cholesterol (mmol/L)		LDL cholesterol (mmol/L)		Triglyceride (mmol/L)	
			
			Mean (SE)	*P*_trend_^a^	Mean (SE)	*P*_trend_^a^	Mean (SE)	*P_t_*_rend_^a^	Mean (SE)	*P*_trend_^a^
Age (years)	<45	353	4.59 (0.04)	<0.01	1.39 (0.02)	<0.01	2.63 (0.04)	<0.01	0.92 (0.04)	<0.01
	45–59	223	5.05 (0.06)		1.32 (0.02)		3.01 (0.05)		1.25 (0.05)	
	≥60	135	5.34 (0.07)		1.32 (0.03)		3.23 (0.06)		1.57 (0.06)	

Percent dense area (%)	<14.71	177	4.93 (0.08)	0.05	1.26 (0.03)	<0.01	2.98 (0.07)	<0.01	1.31 (0.07)	<0.01
	14.71–33.77	179	4.99 (0.06)		1.36 (0.02)		2.95 (0.05)		1.21 (0.05)	
	33.78–59.89	177	4.80 (0.06)		1.37 (0.02)		2.79 (0.06)		1.11 (0.06)	
	≥50.90	178	4.77 (0.07)		1.44 (0.03)		2.73 (0.06)		0.97 (0.06)	

Dense area (cm^2^)	<14.56	177	4.89 (0.07)	0.98	1.30 (0.03)	0.31	2.90 (0.06)	0.94	1.27 (0.07)	0.32
	14.56–32.51	178	4.83 (0.06)		1.38 (0.02)		2.81 (0.05)		1.09 (0.05)	
	32.52–48.01	178	4.92 (0.06)		1.37 (0.02)		2.88 (0.06)		1.12 (0.06)	
	≥48.02	178	4.86 (0.07)		1.37 (0.02)		2.86 (0.06)		1.12 (0.06)	

Body mass index (kg/m^2^)	<18.5	15	4.98 (0.21)	<0.01	1.55 (0.08)	<0.01	2.83 (0.18)	<0.01	0.89 (0.18)	<0.01
	18.5–22.9	318	4.77 (0.05)		1.41 (0.02)		2.75 (0.04)		0.99 (0.04)	
	23.0–24.9	154	4.88 (0.07)		1.35 (0.02)		2.90 (0.06)		1.11 (0.06)	
	≥25	202	5.02 (0.06)		1.25 (0.02)		3.02 (0.05)		1.44 (0.05)	

Smoking status	Never	636	4.87 (0.03)	0.55	1.34 (0.01)	<0.01	2.87 (0.03)	0.22	1.15 (0.03)	0.69
	Ever	69	4.81 (0.10)		1.46 (0.04)		2.76 (0.09)		1.11 (0.09)	

Alcohol drinking	No	379	4.84 (0.04)	0.31	1.32 (0.02)	<0.01	2.87 (0.04)	0.77	1.12 (0.04)	0.41
	Yes	326	4.91 (0.05)		1.40 (0.02)		2.86 (0.04)		1.17 (0.04)	

Physical exercise	No	462	4.86 (0.04)	0.59	1.34 (0.01)	0.38	2.86 (0.03)	0.99	1.16 (0.03)	0.59
	Yes	236	4.90 (0.05)		1.37 (0.02)		2.86 (0.05)		1.13 (0.05)	

Duration of education (years)	<12	222	4.96 (0.07)	0.05	1.30 (0.03)	0.02	2.94 (0.06)	0.10	1.25 (0.06)	0.03
	12–15	332	4.87 (0.05)		1.37 (0.02)		2.85 (0.04)		1.13 (0.04)	
	≥16	155	4.75 (0.07)		1.40 (0.03)		2.78 (0.06)		1.04 (0.06)	

Age at menarche (years)	<12	81	4.74 (0.09)	0.13	1.37 (0.04)	0.28	2.70 (0.08)	0.19	1.29 (0.08)	0.76
	13	108	4.87 (0.08)		1.38 (0.03)		2.91 (0.07)		1.06 (0.07)	
	14	160	4.83 (0.07)		1.36 (0.03)		2.85 (0.06)		1.10 (0.06)	
	15	141	4.84 (0.07)		1.34 (0.03)		2.81 (0.06)		1.22 (0.06)	
	≥16	196	4.96 (0.06)		1.34 (0.03)		2.92 (0.06)		1.13 (0.06)	

Age at first childbirth (years)	<25	194	4.97 (0.06)	0.19	1.33 (0.02)	0.25	2.90 (0.05)	0.55	1.29 (0.06)	0.05
	25–29	358	4.85 (0.04)		1.35 (0.02)		2.85 (0.04)		1.11 (0.04)	
	≥30	90	4.86 (0.09)		1.38 (0.03)		2.86 (0.08)		1.15 (0.08)	

No. live children	None	59	4.98 (0.12)	0.52	1.44 (0.05)	<0.01	2.95 (0.10)	0.60	1.13 (0.11)	0.08
	1–2	395	4.85 (0.05)		1.39 (0.02)		2.83 (0.04)		1.09 (0.04)	
	3–4	164	4.94 (0.07)		1.29 (0.03)		2.94 (0.06)		1.22 (0.07)	
	≥5	73	4.75 (0.12)		1.25 (0.05)		2.76 (0.11)		1.38 (0.11)	

Duration of breast feeding	Never	59	5.01 (0.12)	0.19	1.42 (0.04)	<0.01	2.99 (0.10)	0.38	1.19 (0.09)	0.32
(months)	<6	135	4.91 (0.07)		1.43 (0.03)		2.86 (0.06)		1.08 (0.06)	
	6–11	99	4.82 (0.08)		1.37 (0.03)		2.80 (0.07)		1.09 (0.07)	
	12–23	145	4.87 (0.07)		1.33 (0.03)		2.88 (0.06)		1.17 (0.06)	
	≥24	162	4.79 (0.08)		1.28 (0.03)		2.80 (0.06)		1.20 (0.06)	

Use of oral contraceptive pill	Never	579	4.87 (0.03)	0.97	1.35 (0.01)	0.23	2.85 (0.03)	0.76	1.16 (0.03)	0.32
	Ever	110	4.87 (0.08)		1.39 (0.03)		2.88 (0.07)		1.09 (0.07)	

Hormone replacement therapy	Never	186	5.26 (0.07)	0.61	1.31 (0.02)	0.66	3.19 (0.06)	0.47	1.48 (0.07)	0.75
(among postmenopausal women)	Ever	61	5.19 (0.12)		1.33 (0.04)		3.10 (0.11)		1.43 (0.12)	

Menopause	No	456	4.87 (0.05)	0.93	1.36 (0.02)	0.61	2.85 (0.04)	0.64	1.10 (0.04)	0.12
	Yes	255	4.88 (0.08)		1.34 (0.03)		2.89 (0.07)		1.25 (0.07)	

HDL, high-density lipoprotein; LDL, low-density lipoprotein; SE, standard error.

^a^Assessed by age-adjusted linear regression analysis.

Table 2 shows the relationship between lipid parameters and mammographic density, after adjustment for household effect, twin effect, and potential covariates. In multivariable-adjusted analysis not including BMI, there was a positive association between percent dense area and HDL-C, and the association was stronger in postmenopausal women than in premenopausal women (*P* for interaction <0.05). Although there was no association between HDL-C and dense area when participants were stratified by menopausal status, there was a positive association for all women combined. LDL-C and TG were inversely associated with percent dense area in premenopausal women only, but the interaction between menopausal status and lipid profile (LDL-C and TG) was not significant. However, when the model was additionally adjusted for BMI, the associations of lipid profile with percent dense area and dense area did not persist. We repeated the multivariable analysis separately for obese and nonobese women, but neither dense area nor percent dense area was associated with any lipid parameter (data not shown).

**Table 2.** Multivariable-adjusted associations between lipid parameters and mammographic density

	Multivariable-adjusted^c^ (excluding body mass index)			Multivariable-adjusted^d^ (including body mass index)		
		
	Premenopausal women (*n* = 456)	Postmenopausal women (*n* = 255)	All women (*n* = 711)	Premenopausal women (*n* = 456)	Postmenopausal women (*n* = 255)	All women (*n* = 711)
	β (95% CI)	β (95% CI)^e^	β (95% CI)^f^	β (95% CI)	β (95% CI)^e^	β (95% CI)^f^
Dense area (cm^2^)						
Total cholesterol (mmol/L)	−0.02 (−0.09, 0.06)	0.12 (−0.09, 0.32)	0.04 (−0.03, 0.11)	−0.007 (−0.08, 0.06)	0.12 (−0.11, 0.35)	0.04 (−0.03, 0.12)
HDL cholesterol (mmol/L)	0.15 (−0.03, 0.34)	0.48 (−0.10, 1.06)	0.24^a,g^ (0.04, 0.44)	0.13 (−0.06, 0.32)	0.35 (−0.36, 1.05)	0.20^e^ (−0.003, 0.40)
LDL cholesterol (mmol/L)	−0.02 (−0.10, 0.07)	0.10 (−0.13, 0.34)	0.02 (−0.06, 0.11)	−0.001 (−0.08, 0.08)	0.11 (−0.16, 0.37)	0.04 (−0.05, 0.12)
Triglyceride (mmol/L)	−0.08 (−0.17, 0.01)	−0.02 (−0.22, 0.17)	−0.03 (−0.11, 0.05)	−0.06 (−0.15, 0.03)	0.02 (−0.21, 0.25)	−0.01 (−0.09, 0.07)
Percent dense area, %						
Total cholesterol (mmol/L)	−0.07^a^ (−0.14, −0.01)	0.09 (−0.13, 0.31)	−0.01 (−0.09, 0.06)	−0.04 (−0.10, 0.03)	0.08 (−0.15, 0.32)	0.01 (−0.06, 0.08)
HDL cholesterol (mmol/L)	0.24^b^ (0.07, 0.42)	0.68^a^ (0.06, 1.29)	0.36^b,g^ (0.16, 0.56)	0.13 (−0.04, 0.30)	0.34 (−0.38, 1.06)	0.19 (−0.01, 0.39)
LDL cholesterol (mmol/L)	−0.10^a^ (−0.18, −0.02)	0.07 (−0.17, 0.32)	−0.04 (−0.12, 0.05)	−0.04 (−0.12, 0.03)	0.09 (−0.18, 0.36)	0.00 (−0.08, 0.08)
Triglyceride (mmol/L)	−0.16^b^ (−0.25, −0.07)	−0.10 (−0.31, 0.10)	−0.11^b^ (−0.19, −0.03)	−0.08 (−0.16, 0.01)	−0.02 (−0.25, 0.21)	−0.03 (−0.11, 0.05)

HDL, high-density lipoprotein; LDL, low-density lipoprotein.

^a^*P* < 0.05.

^b^*P* < 0.01.

^c^β (95% confidence interval) was estimated by using a linear mixed model in which random effects (household, twin pair) and fixed effects (age, smoking status, alcohol consumption, physical exercise, education level, number of live children, age at first childbirth, duration of breast feeding, and oral contraceptive pill use) were adjusted for.

^d^Model additionally adjusted for body mass index as a fixed effect.

^e^Model additionally adjusted for use of hormone replacement therapy as a fixed effect.

^f^Model additionally adjusted for menopausal status and use of hormone replacement therapy as fixed effects.

^g^Interaction with menopausal status was significant (*P* < 0.05).

Table 3 shows the relationship between BMI and mammographic density. BMI was inversely associated with percent dense area in both premenopausal and postmenopausal women. Although there was no association between BMI and dense area when participants were stratified by menopausal status, an inverse association was found for all women combined. Additional adjustment for lipid profile did not significantly change the relationship between BMI and mammographic density.

**Table 3.** Multivariable-adjusted associations between body mass index and mammographic density after additional adjustment for lipid parameters

Additionally adjusted lipid parameter (mmol/L)^d^	Dense area (cm^2^)			Percent dense area, %		
	
	Premenopausal women (*n* = 456)	Postmenopausal women (*n* = 255)	All women (*n* = 711)	Premenopausal women (*n* = 456)	Postmenopausal women (*n* = 255)	All women (*n* = 711)
	β (95% CI)^a^	β (95% CI)^b^	β (95% CI)^c^	β (95% CI)^a^	β (95% CI)^b^	β (95% CI)^c^
None	−0.02 (−0.04, 0.002)	−0.05 (−0.11, 0.008)	−0.02 (−0.04, −0.002)	−0.08 (−0.09, −0.06)	−0.11 (−0.17, −0.05)	−0.08 (−0.10, −0.06)
Total cholesterol	−0.02 (−0.04, 0.002)	−0.05 (−0.11, 0.02)	−0.02 (−0.04, −0.003)	−0.07 (−0.09, −0.06)	−0.11 (−0.17, −0.04)	−0.08 (−0.10, −0.06)
HDL cholesterol	−0.02 (−0.04, 0.004)	−0.04 (−0.11, 0.03)	−0.02 (−0.04, −0.002)	−0.07 (−0.09, −0.05)	−0.10 (−0.17, −0.02)	−0.08 (−0.10, −0.06)
LDL cholesterol	−0.02 (−0.04, 0.002)	−0.05 (−0.12, 0.02)	−0.02 (−0.04, −0.003)	−0.07 (−0.09, −0.05)	−0.11 (−0.17, −0.04)	−0.08 (−0.10, −0.06)
Triglyceride	−0.01 (−0.04, 0.007)	−0.05 (−0.12, 0.02)	−0.02 (−0.04, −0.001)	−0.07 (−0.09, −0.05)	−0.11 (−0.18, −0.04)	−0.08 (−0.10, −0.06)

HDL, high-density lipoprotein; LDL, low-density lipoprotein.

^a^β (95% confidence interval) was estimated by using a linear mixed model in which random effects (household, twin pair) and fixed effects (age, smoking status, alcohol consumption, physical exercise, education level, number of live children, age at first childbirth, duration of breast feeding, and oral contraceptive pill use) were adjusted for.

^b^Model additionally adjusted for use of hormone replacement therapy as a fixed effect.

^c^Model additionally adjusted for menopausal status and use of hormone replacement therapy as fixed effects.

^d^Model additionally adjusted for each lipid parameter as a fixed effect.

Table 4 shows additive cross-trait correlations between percent dense area and lipid parameters. In an age-adjusted analysis, HDL-C had a positive genetic correlation with percent dense area and dense area. After additional adjustment for other covariates, the positive genetic correlation between HDL-C and dense area was substantially reduced, while the positive genetic correlation between HDL-C and percent dense area persisted. However, this correlation disappeared when the model was additionally adjusted for BMI.

**Table 4.** Cross-trait correlations^c^ between mammographic density and lipid parameters in the same individual

	Additive genetic correlation			Environmental (unshared) correlation		
		
	Age-adjusted	Multivariable- adjusted^d^ (excluding BMI)	Multivariable- adjusted^d^ (including BMI)	Age-adjusted	Multivariable- adjusted^d^ (excluding BMI)	Multivariable- adjusted^d^ (including BMI)
Correlation with percent dense area						
Total cholesterol	−0.026 (0.073)	−0.025 (0.075)	0.039 (0.080)	−0.094 (0.089)	−0.111 (0.090)	−0.129 (0.089)
HDL cholesterol	0.181 (0.062)^b^	0.175 (0.063)^b^	0.080 (0.066)	0.070 (0.089)	0.071 (0.091)	0.008 (0.091)
LDL cholesterol	−0.052 (0.073)	−0.047 (0.075)	0.034 (0.081)	−0.082 (0.090)	−0.098 (0.091)	−0.110 (0.091)
Triglyceride	−0.270 (0.081)^b^	−0.262 (0.082)^b^	−0.131 (0.091)	−0.063 (0.081)	−0.073 (0.082)	−0.002 (0.083)
Correlation with dense area						
Total cholesterol	0.055 (0.071)	0.028 (0.073)	0.045 (0.074)	0.004 (0.093)	0.019 (0.095)	0.011 (0.096)
HDL cholesterol	0.127 (0.060)^a^	0.095 (0.062)	0.063 (0.062)	−0.029 (0.092)	0.044 (0.095)	0.046 (0.095)
LDL cholesterol	0.037 (0.070)	0.034 (0.073)	0.057 (0.075)	0.034 (0.094)	0.026 (0.097)	0.019 (0.097)
Triglyceride	−0.102 (0.081)	−0.104 (0.083)	−0.061 (0.087)	−0.010 (0.085)	−0.012 (0.087)	−0.003 (0.088)

BMI, body mass index; HDL, high-density lipoprotein; LDL, low-density lipoprotein.

^a^*P* < 0.05.

^b^*P* < 0.01.

^c^Correlation coefficients (standard error) were assessed by bivariate analysis.

^d^Adjusted for age, age^2^, smoking, alcohol consumption, physical exercise, education level, number of live children, age at first childbirth, duration of breast feeding, use of oral contraceptive pill, menopausal status, and use of estrogen replacement therapy.

## DISCUSSION

Evidence indicates that HDL-C increases the risk of breast cancer through its role in cell proliferation in the breast. Although this has not been confirmed in vivo, HDL-C has been shown to affect cell proliferation in culture conditions that may be relevant to carcinogenesis.^4^^,^^5^ Exogenously administered estrogen has been shown to increase HDL-C level,^39^^,^^40^ the extent of parenchymal densities of the breast,^41^^,^^42^ and breast cancer risk.^43^^–^^45^

In premenopausal Canadian women, HDL-C was positively associated with mammographic density, even after accounting for age and BMI,^22^^,^^46^ although the association did not persist when parity, BMI, Apo B, alcohol consumption, skinfold thickness, and urinary malondialdehyde were adjusted for. In the present study, percent dense area was not independently associated with HDL-C, LDL-C, or TG in either premenopausal or postmenopausal Korean women.

When obesity level was not included in the analysis, percent dense area was positively associated with HDL-C and inversely associated with LDL-C and TG. However, these associations disappeared after adjustment for BMI, which suggests that the relations were spurious and probably due to the confounding effect of obesity. When the relationship between percent dense area and HDL-C was examined separately in obese and nonobese women, there was no significant association in either group (data not shown). This suggests that obesity was a confounder rather than an effect modifier.

To further elucidate the relations between BMI, percent dense area, and HDL-C, we investigated the role of HDL-C in the relationship between BMI and percent dense area. We found that HDL-C was inversely associated with BMI. When we examined the relationship between obesity and percent dense area, obesity was inversely associated with percent dense area, and the association was completely unaffected by additional adjustment for lipid parameters. This finding suggests that HDL-C is neither a confounder in the association of obesity with percent dense area nor an intermediate mechanism by which obesity is associated with percent dense area.

Biosynthesis of estrogen differs according to menopausal status, which likely explains why the increase in the risk of breast cancer due to obesity is confined to postmenopausal women.^24^ Because HDL-C level is affected by estrogen replacement and menopausal changes,^47^ we examined whether menopausal status modified the relationship between HDL-C and mammographic density. The BMI-unadjusted association of lipid parameters with percent dense area differed by menopausal status. When we analyzed the combined data after adjustment for menopausal status, the association remained significant. However, the interaction term was not significant, and the association did not persist after adjustment for BMI. Therefore, menopausal status seems to be neither an effect modifier nor a major confounder in the relation between HDL-C and mammographic density.

If mammographic density is an intermediate marker of breast cancer risk, the inverse association between BMI and mammographic density in postmenopausal women is very unusual because BMI is positively associated with breast cancer in postmenopausal women. However, an inverse association between BMI and mammographic density has been consistently observed in many studies,^48^^–^^50^ suggesting that the association between obesity and breast cancer is less likely to be mediated through breast tissue subcomponents that are reflected in mammographic density.

Evaluations of cross-trait genetic correlation between percent dense area and lipid profile have yielded very consistent findings and strongly support the view that the association between percent dense area and HDL-C is not real.

Interestingly, we found that the associations of lipid parameters with dense area and percent dense area differed slightly when BMI was not included in the model, probably because calculation of percent dense area depends on nondense area, which is significantly associated with BMI, while dense area is more independent of BMI. A previous study showed that over one third of the variation in nondense area was explained by BMI, whereas BMI explained a much smaller fraction of the variation in dense area (less than 2%).^48^ These findings suggest that, as compared with percent dense area, dense area is a simpler surrogate marker of proliferation in breast tissue, as it is less influenced by BMI level.

Several limitations in this study should be considered. We were unable to investigate the direct relationship between HDL-C and breast cancer in Korean women. Therefore, an association between HDL-C and breast cancer cannot be completely excluded, even though findings from our study on lipid and percent dense area do not support a role for HDL-C as a risk factor in breast cancer. In a previous cohort study of postmenopausal Korean women, we observed that serum T-C level was not associated with breast cancer.^51^ In the present study, a high cholesterol level was initially associated with a greater risk of breast cancer; however, the association disappeared when the model was additionally adjusted for BMI. The relation between HDL-C and breast cancer was not examined in the cohort study because data on HDL-C were not available. Recently, a large case-control study in Korean women showed that high HDL-C had a protective effect against breast cancer among nonobese premenopausal women and that BMI modified the association between HDL-C and breast cancer.^12^ However, the HDL-C level of the breast cancer cases was measured after a diagnosis of breast cancer in the case-control study, and reverse causation cannot be completely ruled out. Further study will be needed to clarify the issue.

Mammographic density is a relative value, and the characteristics of X-ray attenuation in connective and epithelial tissue (dense area) and fat tissue (nondense area)^15^^,^^52^ may reflect cell proliferation activity in the breast. The known association of mammographic density with breast cancer has led to its use as a surrogate marker in research evaluating the mechanism by which factors that influence cell proliferation in breast tissue increase the risk of breast cancer. However, mammographic density may have limitations as an intermediate marker of breast cancer risk, because not all risk factors for breast cancer have the same association with mammographic density.^52^ We used a single measurement of lipid parameters, and substantial biological variation might have biased results. In addition, mammographic density and lipid parameters change during the menstrual cycle.^53^^,^^54^ However, we could not fully assess the menstrual cycle phase in premenopausal women and were thus unable to perform mammograms and blood testing during the same menstrual cycle phase in premenstrual women. The association between HDL-C and other lipid parameters with mammographic density may differ by ethnic group, given that Asian women differ greatly from Western women in HDL-C and obesity level. Therefore, findings from our study may not be generalizable to other ethnic populations.

In conclusion, there was no independent association between HDL-C, LDL-C, or TG and mammographic density in Korean women. These findings suggest that HDL-C is unlikely to increase the risk of breast cancer, particularly via changes in breast parenchyma that are reflected in mammographic density. In addition, studies that use mammographic density as an intermediate marker of breast cancer risk should control for BMI.
